# Mid-Infrared Tunable Resonant Cavity Enhanced Detectors

**DOI:** 10.3390/s8095466

**Published:** 2008-09-04

**Authors:** Niels Quack, Stefan Blunier, Jurg Dual, Ferdinand Felder, Martin Arnold, Hans Zogg

**Affiliations:** 1 Institute of Mechanical Systems, Department of Mechanical and Process Engineering, ETH Zurich. Center of Mechanics, Tannenstrasse 3, ETH Zentrum CLA J35, CH-8092 Zürich, Switzerland; Phone: +41 44 632 35 63, Fax: +41 44 632 11 45; E-mail: niels.quack@imes.mavt.ethz.ch; stefan.blunier@imes.mavt.ethz.ch; dual@imes.mavt.ethz.ch; 2 Thin Film Physics Group, Technopark, ETH-Trakt PFA J25, Technoparkstrasse 1, CH-8005 Zürich, Switzerland. Phone: +41 44 633 79 55, Fax: +41 44 633 18 61; E-mail: ffelder@phys.ethz.ch; zogg@phys.ethz.ch

**Keywords:** RCED, MEMS, micromirror, Fabry-Perot, mid infrared, micro spectrometer

## Abstract

Mid-infrared detectors that are sensitive only in a tunable narrow spectral band are presented. They are based on the Resonant Cavity Enhanced Detector (RCED) principle and employing a thin active region using IV-VI narrow gap semiconductor layers. A Fabry-Pérot cavity is formed by two mirrors. The active layer is grown onto one mirror, while the second mirror can be displaced. This changes the cavity length thus shifting the resonances where the detector is sensitive. Using electrostatically actuated MEMS micromirrors, a very compact tunable detector system has been fabricated. Mirror movements of more than 3 μm at 30V are obtained. With these mirrors, detectors with a wavelength tuning range of about 0.7 μm have been realized. Single detectors can be used in mid-infrared micro spectrometers, while a detector arrangement in an array makes it possible to realize Adaptive Focal Plane Arrays (AFPA).

## Introduction

Tunable Resonant Cavity Enhanced Detectors (RCED) allow one to realize compact spectrometers in the mid-infrared (mid-IR) spectral range [1]. The photodetector is placed inside a Fabry-Pérot cavity. Incoming radiation is reflected multiple times within the cavity and a standing wave pattern forms. The detector is highly sensitive almost exclusively at those resonances. The peak wavelengths of the resonances depend on the distance between the two mirrors of the cavity. Displacing one of the cavity mirrors changes the cavity length and thus the peak detection wavelengths. The mirror displacement can be achieved with an external piezo-actuated mirror [2] or an integrated Micro-Electronic-Mechanical System (MEMS) micromirror [3, 4]. Mid-IR RCED with a fixed cavity length have already been reported [5].

**Figure 1.**
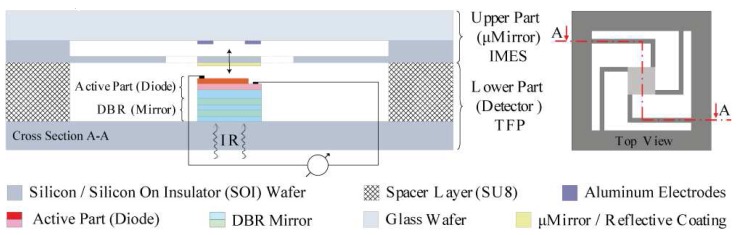
Tunable RCED working principle. Left: cross section with micromirror (upper part) and detector (lower part). Right: top view showing micromirror and mirror suspensions.

With an integrated MEMS mirror, a very compact detector system can be built. Figure 1 shows a schematic representation of such an integrated tunable RCED. The device can be divided in two parts: a lower part with the detector containing the fixed Distributed Bragg Reflector (DBR) and the p-n photo diode and the upper part with the movable MEMS mirror.

The DBR consists of few quarter wavelength layer pairs with alternating high and low refractive index layers grown by molecular beam epitaxy. They are fabricated using lead chalcogenide (IV-VI narrow gap semiconductor) materials and EuTe for high and low index respectively. The index contrast is very high, resulting in near 100% reflectivity with a few quarter wavelength pairs over a broad spectral band. The active region is formed by a p(PbTe)–n^+^(PbSrTe:Bi) heterojunction grown on the DBR. In order to select one detection resonance only, a PbSrTe buffer layer is included. Due to its alloying the cut-off is shifted towards shorter wavelengths which are absorbed, while being transparent for the design wavelengths. The tunable range is therefore confined by the cut-off wavelength of the buffer and the cut-off of the photodiode [5].

The upper part contains the movable MEMS structure. It is fabricated in the highly doped device layer of an SOI wafer and bonded anodically to a glass wafer, where the actuation counter electrodes are placed. A reflective gold coating evaporated on the suspended silicon membrane serves as mirror. The detector including mirrors, electrostatic actuation and the optical cavity has a thickness of only about 30 μm.

**Figure 2.**
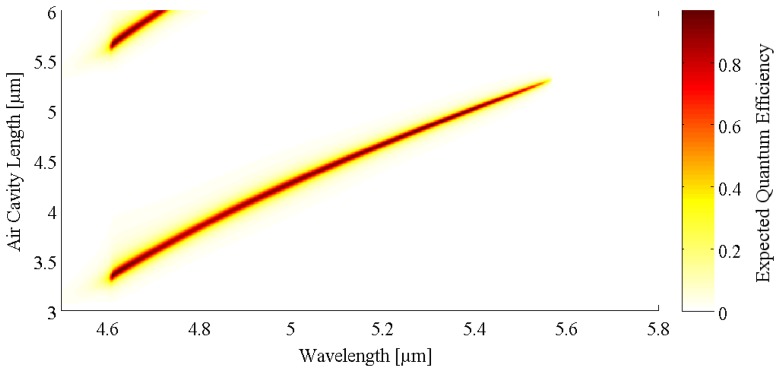
Detector quantum efficiency simulated for perpendicular incident radiation at 100 K using the transfer matrix method. Displacing the movable MEMS mirror allows changing the air cavity length and thus the detection wavelength. At a certain mirror position, the detector is sensitive at a single narrow wavelength band only. Shorter wavelengths are absorbed in a buffer layer while longer wavelengths are above the designed cut-off of the diode.

The width of the resonance peaks is determined by the reflectance of the mirrors and the absorption within the cavity (the finesse of the cavity), as well as the order of the resonance [1]. The detector order configuration is chosen by the initial distance of the movable mirror, additionally limited by the material within the cavity. In Figure 2 the simulation of the response of a RCED with a similar setup as presented below for the detector employing the comb drive actuated micromirror (R_1_ ∼ 90%, R_2_ = 99%, reduced absorber thickness) is shown. With this configuration, a single detection peak can be obtained and the detection wavelength can be shifted from 4.6 μm to 5.5 μm for a mirror movement range of 2 μm. With the simulation the strength of the tunable RCED principle becomes apparent; the cavity effect allows both field enhancement and at the same time to shift the detected wavelengths.

## 2. Movable MEMS Mirrors

For a displacement range of about 2 μm of the movable micromirror different actuation methods can be applied. Realized detector systems are presented using piezoelectric actuation [2], electrostatic actuation in a parallel plate configuration [6] and electrostatic actuation in a comb drive configuration [4]. Integration of the detector and the mirror is hereby possible in a much higher degree for the MEMS micromirrors than for the piezoelectrically actuated micromirrors.

### 2.1. Parallel Plate Electrostatic Actuation

**Figure 3.**
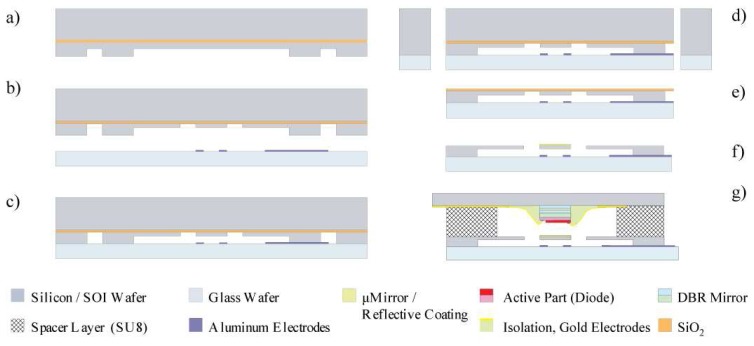
Tunable RCED fabrication process: two successive silicon DRIE steps to form a) the electrostatic actuation gap and b) the mirror and suspension. Glass wafer preparation with counter electrodes and c) anodic bonding, followed by d) dicing and e) handle layer and buried oxide removal. f) Reflective coating deposition and g) integration with the detector part, resulting in the complete tunable RCED device as shown in Figure 1.

The fabrication of a micromirror actuated by parallel plate electrostatic actuators is based on a process using Silicon on Insulator wafers (SOI, Device Layer 20 μm/Buried Oxide Layer 4 μm/Handle Layer 300 μm) as shown in Figure 3. The highly doped device layer is structured in two steps by Deep Reactive Ion Etching (DRIE). The first etch step of ∼10 μm defines the electrostatic actuation gap. The second etch step defines the mirror and the suspension legs in the remaining device layer. The structured SOI wafer is bonded anodically to a glass wafer containing the aluminum counter electrodes. The wafers are then diced and for the individual devices, the handle and the buried oxide layer are removed by dry etching. A reflective gold coating is then evaporated onto the movable mirror using a shadow mask and integration with the detector part follows, resulting in the complete tunable RCED device as shown in Figure 1.

**Figure 4.**
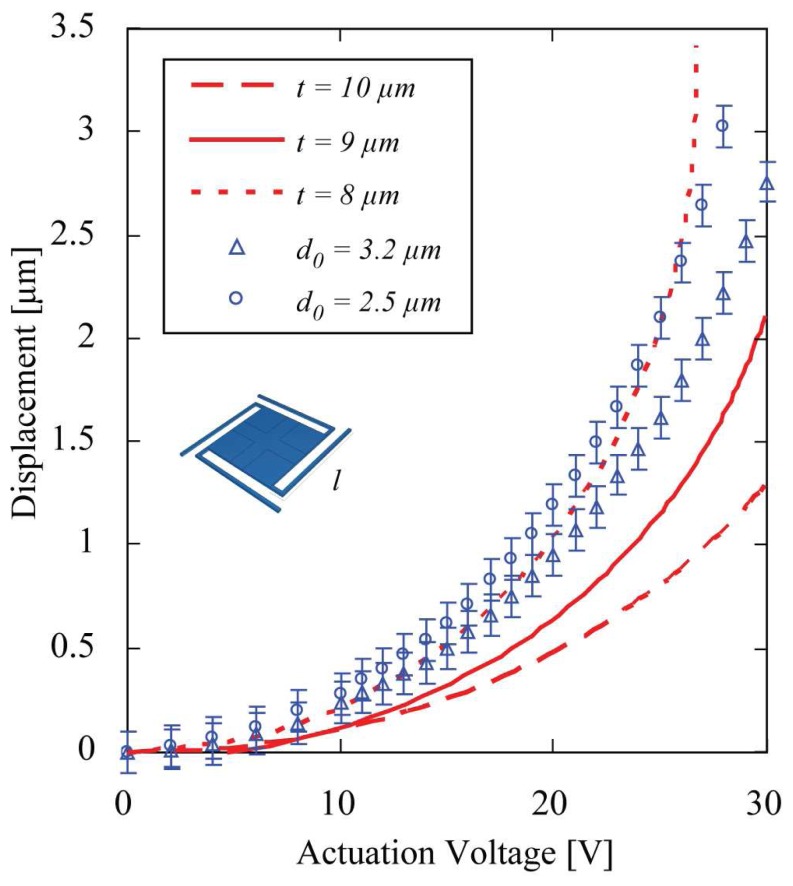
Finite Element simulations (lines) and measurements for different gold coated devices (data points) of the mirror displacement vs. applied voltage on the electrostatic actuators. Mirror square length is *l* = 400 μm and thickness about *t* = 9 μm. The differences are due to variations in thickness and different initial displacements d_0_.

The displacement of the micromirror depends quadratically on the applied voltage. The suspension geometry defines the elastic constant and thus the applied voltage needed for a certain displacement. Finite element simulations show, that for a mirror with a thickness of 9 μm, a size of 400 μm × 400 μm, and a total length of the folded suspension of 1370 μm, an actuation voltage of about 30 V is needed to obtain a displacement of 3 μm. The fabricated mirrors show displacements of more than 2 μm, using actuation voltages below 30 V (Figure 4). However, the experimental results differ slightly from the simulated ones. One reason for this difference are the thickness variations of the mirror samples batch fabricated on one wafer due to the fabrication process. The plot in Figure 4 shows, that the displacements of the measured micromirrors lie within bounds given by thickness variations of 1 μm around the intended thickness of 9 μm. Thickness variations over one single micromirror are given by the second DRIE step and lie in the order of tenth of nanometers over a 400 μm × 400 μm mirror device. Secondly, the mirrors suffered from curvature due to released internal stresses, resulting in an initial mirror center displacement d_0_ without applied voltage. The initial mirror elevation d_0_ was measured to be maximum about 3 μm in the mirror center over the total mirror length (400 μm), which corresponds to a radius of curvature of about 0.16 m. The curvature was observed directly after release of the device, before applying the reflective coating. The deposit of a reflective gold coating with a thickness of 60 nm on the 9 μm thick silicon mirror membrane does not change the curvature significantly. We thus believe, that these internal stresses are due to the thermal treatment during the bond process and to initial residual stresses in the device layer of the SOI wafer. Temperature adjustments during the anodic bonding process, a stiffness increase of the mirror membrane or the deposit of a metal layer of adequate thickness on the mirror are possible strategies to reduce or compensate the curvature in a next generation. This is of importance in order to increase the optical performance of the detector system, as the curvature degrades the finesse of the optical cavity.

**Figure 5.**
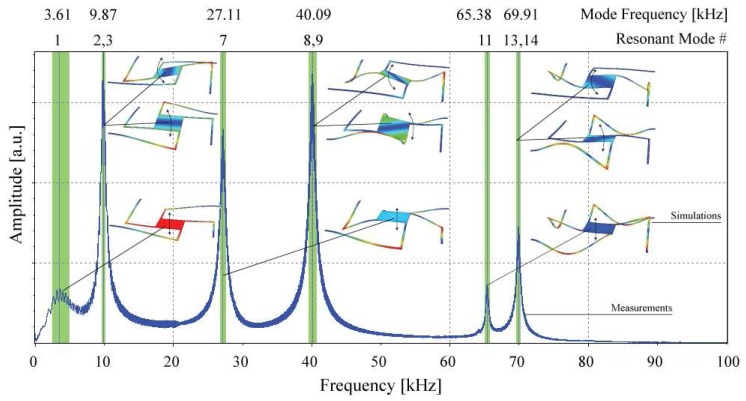
Measurements of the mirror resonance frequencies and schematic representation of the different resonance modes obtained from the Finite Element Model frequency analysis. The modes which are not represented were not measured as their movement lies in the mirror plane.

The displacements measured characterize the static mirror position for an applied voltage. With mechanical resonances in vertical direction however, the micromirrors can be used in a resonant operating mode in a tunable RCED, an adequate readout mechanism provided. Other mode shapes could moreover be of interest for various applications, such as tilting mirrors for bar code readers or displaying applications. The micromirror dynamic properties at atmospheric pressure with electrostatic actuation of the devices are depicted in Figure 5. The measured curve shows the frequency response to an applied periodic chirp signal of 30 V amplitude performed on a device with 400 μm × 400 μm mirror size, suspension length 1370 μm and a thickness of 9 μm. The figure illustrates the resonant mode shapes and the frequencies which they appear at. With the measurement setup used, only movements in vertical direction were characterized. The frequencies of the resonance modes with a movement in vertical direction were measured and compared to the frequency analysis results obtained from the Finite Element Model. The numbering of the resonant modes corresponds to their appearance with increasing frequency in the FEM simulations. The resonance modes obtained from simulations appear in the same sequence as the measured ones so that each measured vertical mode could be identified and assigned to a vertical mode obtained from the simulation results. However, simulated frequencies were typically some per cent higher, which is probably due to the fact that the FEM model did not take into account squeeze-film air damping effects in the actuation gap. The resonance mode pairs 2/3, 8/9 and 13/14 appeared at single frequencies due to the mirror design symmetry.

### 2.1. Comb-Drive Actuation

**Figure 6.**
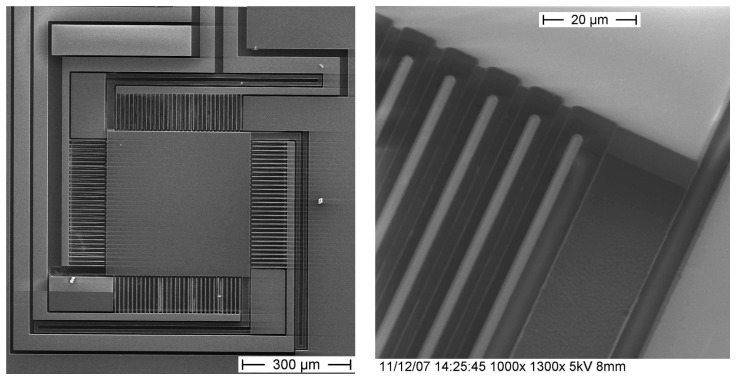
SEM images of fabricated comb-drive actuated micromirrors. Left: overview of a fabricated micromirror. Right: Close-up combs and suspensions. The lower suspensions and combs on the mirror appear in dark (low height), while the elevated regions of the mirror surface and fixed combs appear bright (high height).

An alternative to the parallel plate electrostatic actuation is a vertically actuated comb-drive mechanism. The comb drives are hereby fabricated in the device layer of a SOI wafer (Device Layer 20 μm/Buried Oxide Layer 4 μm/Handle Layer 300 μm). The thickness of the suspension beams and the actuation combs attached to the mirror differs from the thickness of the mirror and the fixed combs. In this way, the mirror plate can be fabricated much stiffer than the suspensions. Deformations due to internal stresses and due to the actuation are mainly induced into the suspension and not into the mirror plate in such a configuration. This has the advantage, that the mirror surface curvature is drastically reduced. The actuation mechanism is based on the asymmetry of the electric field which builds up when an actuation voltage between the movable mirror combs and the fixed combs is applied. The electric field causes the movable (low height) combs to move vertically towards the center of the fixed (high height) combs. Figure 6 shows a SEM image of a fabricated micromirror, where the separation of the low height combs (dark) from the high height combs (bright) is visible. The realization of such vertically moving comb drive actuated micromirrors has been achieved with a delay mask process based on a fabrication process presented by Noell *et al.* [7]. Mirrors with geometries comparable to the parallel plate actuated micromirror with mirror square length *l* = 300 μm and *l* = 400 μm have been realized.

**Figure 7.**
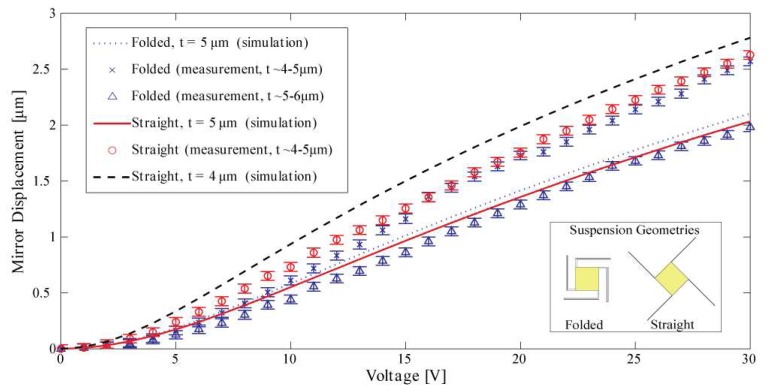
Simulations and measurements of mirror displacements for actuation voltages applied at all comb drives simultaneously. The discrepancies between the theoretical and measured curve have their origin in thickness variations of the suspensions due to the fabrication process.

Displacements of 2.5 μm with an actuation voltage of 30V have been measured as predicted by Finite Element simulations as shown in Figure 7. The simulation results were obtained by first calculating the electrostatic energy for a certain displacement of the micromirror and then deriving the energy with respect to the displacement in order to obtain the electrostatic force necessary for the displacement. In an equilibrium position, the electrostatic force has to be equal to the mechanical force of the spring. Solving this system allows to obtain the displacement as a function of the applied voltage. For the single crystalline silicon an orthotropic elastic material was assumed. The mirror square length is *l* = 400 μm, comb drive finger gap is 3 μm, finger width 2 μm, length 100 (120) μm, suspension width 10 (5) μm and length 780 (1340) μm for the straight (folded) suspension.

**Figure 8.**
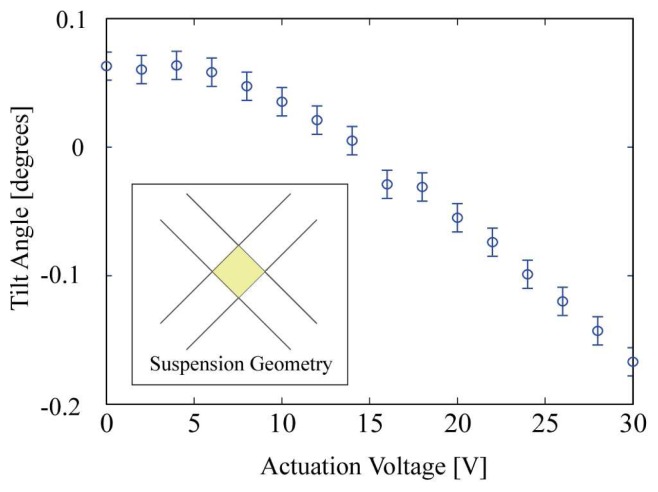
Mirror tilting by applying a potential difference between two opposite comb drives.

In order to account for a non parallel alignment of the movable mirror with respect to the DBR mirror, the movable mirror can be tilted by applying different voltages to the different comb drives. By applying a voltage difference on two opposite comb drives while keeping the other comb drives grounded, a total angle variation of 0.23 degrees has been achieved as shown in Figure 8. This would compensate a thickness variation of the SU8 layer of up to 30 μm over the 7 mm wide spacer layer. This is far above the usually encountered thickness variations in such thin films which use to be well below 1 μm.

Due to the fabrication process and in contrast to the parallel plate actuated micromirror, the comb drive actuated micromirror exhibits after release a curvature towards the diode. Radius of curvature of the micromirror is about 1.2 m with a roughness of 2 nm (root mean square, corrected for the spherical bow). The thickness variations for these micromirrors are minimal as they are given by the polishing step by the manufacturer of the SOI wafer.

The first two mechanical resonance modes with a vertical movement for the comb drive actuated micromirrors lie below 100 kHz and show a parallel mirror movement and a tilting movement, similar to the first two modes of the parallel plate actuated micromirror. These mechanical resonance modes depend strongly on the geometry of the mirror and the suspensions, whereby the thickness of the suspensions has a major influence. Resonance frequencies can be designed around 10 kHz for the vertical parallel mirror movement.

## 3. Fixed mirror and detector

**Figure 9.**
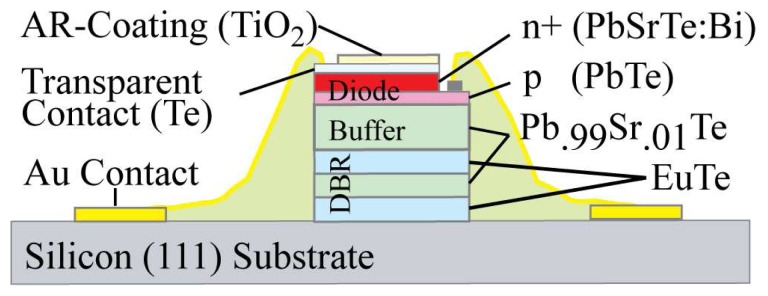
Detailed schematic representation of the detector part layout as shown in the lower part of Figure 1, including the Distributed Bragg Reflector (DBR), the buffer layer and the photosensitive diode.

On the detector side, a fixed mirror, the active diode with an antireflective coating and a transparent contact are grown onto a Si (111) substrate. The fixed mirror of the tunable RCED is formed by a DBR with up to 1.5 pairs of alternating layers with high (Pb_0.99_Sr_0.01_Te, n ∼ 6) and low (EuTe, n ∼ 2.4) refractive indices. With the high contrast in refractive index, 1.5 pairs mirror reflectivity is above 90% for wavelengths from 4 μm to 6 μm. The mirror is followed by a 3 μm buffer layer Pb_0.99_Sr_0.01_Te, which absorbs wavelengths below 4.7 μm (100 K) in order to select a single detection peak. In a second configuration used with the Comb Drive, the buffer layer was grown first, followed by the DBR, for a reduced cavity length. The active region is formed by a p(PbTe)–n^+^(PbSrTe:Bi) diode. Te excess accounts for p-type semiconductor while Bi doping ensures n^+^ type conductivity. The absorbing layer is only 0.3 μm thin. When compared to a bulk photodetector, the thin layer has a reduced volume where noise due to Shockley-Read generation and recombination of charge carriers can originate, which results in a drastically increased sensitivity of the device [2]. The Te transparent contact ensures the n^+^ side diode contact and an anti-reflective TiO_2_ coating on top minimizes unwanted reflections at the diode / air gap interface. A p-n junction with transparent contacts has to be used for tunable detectors, while, in contrast, a metal as part of the photovoltaic detector and acting as a mirror was employed in the RCED with a fixed cavity length [5]. The schematics of the detector part are shown in Figure 9.

## 4. Tunable Mid-Infrared Detector Results and Discussion

Tunable detectors for the mid-infrared have been realized with three different actuation mechanisms: With a mirror mounted on an external piezo-actuator and with electrostatically actuated MEMS micromirrors in a parallel plate configuration and in a comb drive configuration. In Table 1 the characteristics of each setup are compared. In all cases the top gold mirror had a reflectance of ∼99%.

**Figure 10.**
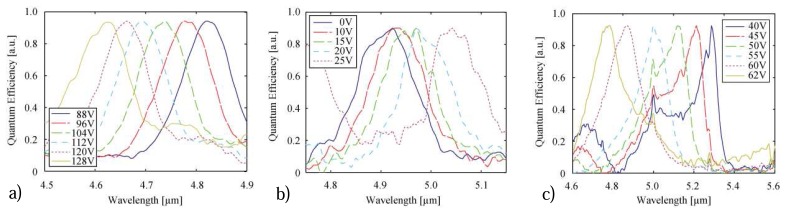
Measured spectral responses for micromirrors actuated with (a) piezoelectric actuators, (b) parallel plate electrostatic actuators, and (c) comb drive actuators for different actuation voltages corresponding to different positions of the movable mirror. The tuning range of the detectors depends on the initial air gap between the movable mirror and the diode and on the mirror displacement. The detection peak irregularity for the MEMS micromirrors is due to a reduced finesse of the cavity.

The measured spectral detector response for different actuation voltages, corresponding to different movable mirror positions, is shown in Figure 10 for each tunable detector type. The recorded signals show that the detection signal can be tuned in a range between 4.5 μm and 5.3 μm, depending on the actuation type and configuration of the lower detector part. Absolute quantum efficiency was not measured; however previous reported monolithic RCED exhibited a quantum efficiency of 35% [5].

As the MEMS parallel plate actuated mirror moves away from the DBR, the air gap increases and the detection wavelength is shifted to longer wavelengths. For the piezo-actuated mirror and the comb drive actuated MEMS mirror, the movement towards the DBR, the air gap decreases and the detection wavelength is shifted to shorter wavelengths.

The detection peaks of the piezo-actuated mirror are of a rather regular shape. Due to the advanced technological process needed for fabrication, the detected signals for the MEMS mirrors are less regular. Eventual mirror plate defects like curvature of the mirror plate, mirror surface roughness and departure from parallelism to the DBR mirror may degrade the cavity. In case of the MEMS setups, a slight misalignment of the actuated mirror and of the reflective coating further deteriorate the resonance peak. Besides an increased peak width, such a misalignment produces a secondary unwanted resonance which is independent of the mirror position. The curves shown in Figure 10 c) were corrected for this particular background by subtracting the response of the secondary cavity. The correction has no influence on the already deteriorated peak width.

The detectors were characterized at operating temperatures between 90 K and 135 K (The detectors can be used up to > 220K or even RT). Du to the temperature dependent bandgaps of the photodiode and the buffer layer, the tunable region is shifted. Contrary to most other semiconductors, the cut-off wavelengths of lead salts increase with decreasing temperature.

The characteristics for the different detectors are represented in Table 1. With the integrated MEMS actuated versions, the air gap can be drastically reduced. This leads to a lower order configuration of the detector, resulting in an increased free spectral range. The initial air gap distance can be chosen for a single detector during the process by adjusting the SU8 spacer layer thickness. The large optical cavity length in the first two setups is due to the buffer layer being within the cavity. Originally necessary for improved crystal quality of the active layer, it can now be grown outside of the cavity due to advances in crystal growth.

There are distinct variations in FWHM. For the Piezo setup, the limiting factor is the fine tuning of the parallelism of the two mirrors. The increased FWHM of the MEMS micromirrors on the other hand, especially of the Comb Drive sample, is mainly caused by a misalignment of the gold layer on the micromirror, as well as a misalignment of the mirror and the diode. This is a technological issue which can be solved by slight changes in process. Additionally the finesse and therefore the FWHM depends on the absorption within the cavity.

## 5. Conclusions

The first tunable Resonant Cavity Enhanced Detectors for the mid-infrared spectral range are reported. The fabricated devices show tuning ranges from 4.6 μm to 5.3 μm at 90K. Due to the low noise, the devices can be operated at temperatures up to 250K, which can be achieved with thermoelectric cooling. With an improved design of the presented detector system, a tuning range from 4 μm to 5.5 μm is possible. Furthermore, by improving the cavity finesse, for example with an even thinner absorbing layer, the detector spectral bandwidth can be reduced.

Especially advantageous in the RCED configuration compared to a passive band-pass filter in front of a conventional broadband photodetector [8] is the reduced noise. This is due to the very thin active detector layer (0.3 μm) generating less noise, while the high optical intensity due to the resonance effect in the optical cavity still yields very high quantum efficiency.

These detector systems can then be used for miniaturized spectroscopy systems, hyperspectral imaging or thermography applications.

## Figures and Tables

**Table 1.** Characteristic data for detectors using piezo-actuation or MEMS electrostatic actuation in a parallel plate or comb drive configuration as tuning actuation mechanisms.

	**Piezo-Actuation**	**MEMS Parallel Plate**	**MEMS Comb Drive**

Approximately DBR Reflectance	80%	80%	90%
Calculated Finesse (w/o absorber)	10 (27)	10 (27)	13 (55)
FWHM δλ [nm] (δλ/λ)	∼75 (1.5%)	∼100 (1.9%)	∼150 (3%)
Maximum Tuning Voltage [V]	128	25	62
Maximum Displacement [μm]	6	4.5	2.5
Optical Cavity Length [μm]	44 - 50	28.5 - 33	14.5 - 17
Minimum Air Gap [μm]	20	4	10
Tuning Range Δλ [μm]	4.55 - 4.85	4.85 - 5.15	4.7 - 5.4
